# A population-based dataset concerning predictors of willingness to get a COVID-19 vaccine in Iran

**DOI:** 10.1016/j.dib.2021.107459

**Published:** 2021-10-08

**Authors:** Amir H. Pakpour, Rafat Yahaghi, Safie Ahmadizade, Razie Fotuhi, Elham Taherkhani, Mehdi Ranjbaran, Zeinab Buchali, Chung-Ying Lin, Mark D. Griffiths, Anders Broström

**Affiliations:** a Social Determinants of Health Research Center, Research Institute for Prevention of Non-Communicable Diseases, Qazvin University of Medical Sciences, 3419759811, Qazvin, Iran; b Department of Nursing, School of Health and Welfare, Jönköping University, Jönköping, Sweden; c Institute of Allied Health Sciences, College of Medicine, National Cheng Kung University, Tainan, Taiwan; d International Gaming Research Unit, Psychology Department, Nottingham Trent University, Nottingham, UK

**Keywords:** COVID-19, Intention, Iran, Survey, Vaccination

## Abstract

The global issue of preventing the spread of COVID-19 is challenging. One of the most efficient ways to control the pandemic is to have a full coverage of COVID-19 vaccination. Therefore, this paper collected survey data to understand the intention and willingness of COVID-19 vaccination uptake in Qazvin, Iran. With the use of a paper-and-pencil method and multistage stratified cluster sampling, research personnel approached and interviewed a representative sample of adults in Qazvin (*n* = 10843) between February 19 and April 9, 2021. The survey asked questions regarding sociodemographic information, fear of COVID-19, perceived COVID-19 infectability, perceived behavioral control over COVID-19 vaccination, subjective norm of COVID-19 vaccination, attitude towards COVID-19 vaccination, and intention to get COVID-19 vaccinated. The data collected from this survey were analyzed using descriptive statistics, which were carried out using the IBM SPSS version 17.0.

## Specifications Table

**Table:** 

Subject	Infectious diseases and public health
Specific subject area	Health behaviors and psychology
Type of data	Tables
How data were acquired	Data were collected using paper-and-pencil method where interviewee completed the form. A copy of the survey is included as Supplementary File.
Data format	Raw, analyzed
Parameters for data collection	The target population was adult general population in Qazvin, Iran (n=10843). The survey questions include basic sociodemographic information, fear of COVID-19, perceived COVID-19 infectability, perceived behavioral control over COVID-19 vaccination, subjective norm of COVID-19 vaccination, attitude toward COVID-19 vaccination, and intention to get COVID-19 vaccinated.
Description of data collection	The data were collected using paper-and-pencil method and multistage stratified cluster sampling. Several interviewers who were well trained approached eligible participants to complete the survey questions. The participants were a representative sample in Qazvin.
Data source location	The data were collected by the Social Determinants of Health Research Center, Qazvin University of Medical Sciences – Iran.
Data accessibility	Repository name: Harvard Dataverse Data identification number: doi: 10.7910/DVN/IETC88 Direct URL to data: https://dataverse.harvard.edu/dataset.xhtml?persistentId=doi:10.7910/DVN/IETC88

## Value of the Data

• This dataset is useful because it comprises data from a largescale study that includes a representative sample in Qazvin Iran to assess factors related to willingness of COVID-19 vaccination uptake, including fear of COVID-19, perceived COVID-19 infectability, attitude toward COVID-19 vaccination, subjective norm of COVID-19 vaccination, and perceived behavioral control over COVID-19 vaccination. Moreover, intention toward getting COVID-19 vaccinated was assessed in this dataset.
• The dataset can benefit the following personnel: researchers in communicable disease, health behavior, health promotion, health psychology, public health, and epidemiology because the findings provide information and knowledge regarding general population's attitudes, subjective norm, perceived behavioral control, and intention toward COVID-19 vaccination. Moreover, the information of perceived COVID-19 infectability was collected in this dataset. Therefore, different health disciplines can use the data for health promotion and education to advocate and elevate general population's willingness to get COVID-19 vaccinated.
• The dataset is useful for academic researchers who would like to understand the underlying psychological mechanisms of intention to uptake COVID-19 vaccination. More specifically, the present study's results can be compared with relevant studies from other countries to examine whether the psychological mechanisms can be applied to different countries. Moreover, systematic review and meta-analysis studies can be conducted in the future.
• The findings from the present dataset may assist the authorities, including government and health policymakers, in decision making by using scientific evidence to develop and implement COVID-19 vaccination uptake guidelines.

## 1 Data Description

With the use of a representative sample, the present dataset provides insightful and useful information regarding COVID-19 vaccination uptake. The information was collected via paper-and-pencil survey data in a general population of Qazvin, Iran. The present dataset included survey data from 10843 adults to understand their intention to get COVID-19 vaccinated. Moreover, potential factors that can explain intention to get COVID-19 vaccinated were collected. These factors were derived from two important health behavior theories: Theory of Planned Behavior [1] and Protection Motivation Theory [2]. More specifically, the factors derived from the Theory of Planned Behavior include attitude toward COVID-19 vaccination (i.e., how an individual evaluates the value of COVID-19 vaccination), subjective norm of COVID-19 vaccination (i.e., how an individual perceives others’ opinions toward COVID-19 vaccination), and perceived behavioral control over COVID-19 vaccination (i.e., how an individual has confidence in getting COVID-19 vaccinated) [3,4]. The factors derived from the Protection Motivation Theory include fear of COVID-19 and perceived infectability (i.e., how an individual perceives the possibility of getting COVID-19 infection) [5,6]. The English version of the survey questionnaire is attached as a supplementary file. With understanding the intention of COVID-19 vaccination uptake (i.e., how an individual is willing to get COVID-19 vaccinated), herd immunity may be achieved [7]; subsequently, psychological distress induced by the COVID-19 may be somewhat lessened [8], [9], [10], [11]. Table 1 illustrates the participants’ sociodemographic characteristics. Tables 2 and 3 shows the distributions of responses related to the factors in the Theory of Planned Behavior. More specifically, Table 2 presents participants’ attitude toward COVID-19 vaccination; Table 3 presents participants’ subjective norm of COVID-19 vaccination, perceived behavioral control over COVID-19 vaccination, and intention to get COVID-19 vaccinated. Tables 4 and 5 demonstrates the distributions of responses related to the factors in the Protection Motivation Theory. More specifically, Table 4 presents participants’ fear of COVID-19 and Table 5 presents participants’ perceived COVID-19 infectability.

**Table 1:** Distribution of responses in relation to socio-demographic variables.

Socio – demographics	Frequency	Percentages
**Age group; Mean ± SD = 35.54 ± 12.00 years**		
18–29 years	3431	31.6
30–39 years	3820	35.2
40–49 years	2327	21.5
50–59 years	815	7.5
60 years and above (elderly)	438	4.0
**Gender**		
Male	4092	37.7
Female	6751	62.3
**Educational status**		
No formal education	352	3.2
Primary school (up to 6)	986	9.1
Secondary school (7 to 9)	1540	14.2
Higher school (10 to 12)	974	9.0
Diploma	2761	25.5
University	4230	39.0
**Divisional residence**		
Qazvin	4787	44.1
Takestan	1336	12.3
Avaj	307	2.8
Alborz	1145	10.6
Buin Zahra	988	9.1
Abyek	753	6.9
Eqbaliyeh	453	4.2
Mohammadiyeh	872	8.0
**Administrative residence**		
Rural	2656	24.5
City	8187	75.5
**Marital status**		
Unmarried	2751	25.4
Married	8092	74.6
**Having a child**		
Yes	3884	35.8
No	6959	64.2

**Table 2:** Distribution of responses in relation to Attitude toward COVID-19 vaccination.

Attitude toward COVID-19 vaccination		Frequency	Percentages
For me, getting the COVID-19 vaccination would be …	extremely bad (1)	439	4.0
	(2)	332	3.1
	(3)	3081	28.4
	(4)	2638	24.3
	extremely good (5)	4304	39.7
	extremely undesirable (1)	413	3.8
	(2)	530	4.9
	(3)	2733	25.2
	(4)	3191	29.4
	extremely desirable (5)	3928	36.2
	extremely unimportant (1)	374	3.4
	(2)	403	3.7
	(3)	2788	25.7
	(4)	3421	31.6
	extremely important (5)	3802	35.1
	extremely useless (1)	439	4.0
	(2)	451	4.2
	(3)	2863	26.4
	(4)	3473	32.0
	extremely useful (5)	3567	32.9
	extremely unfavorable (1)	779	7.2
	(2)	718	6.6
	(3)	2754	25.4
	(4)	2989	27.6
	extremely favorable (5)	3568	32.9
	extremely harmful (1)	618	5.7
	(2)	587	5.4
	(3)	2446	22.6
	(4)	3339	30.8
	extremely beneficial (5)	3817	35.2

**Table 3:** Distribution of responses related to subjective norms, perceived behavioral control and intention.

		Frequency	Percentages
Most people who are important to me would want me to get a COVID-19 vaccination	Strongly Disagree	658	6.1
	Disagree	747	6.9
	Neutral	2419	22.3
	Agree	3052	28.1
	Strongly Agree	3935	36.3

Most people who are important to me would think I should get a COVID-19 vaccination	Strongly Disagree	608	5.6
	Disagree	708	6.5
	Neutral	2414	22.3
	Agree	3179	29.3
	Strongly Agree	3898	35.9

Whether or not I get a COVID-19 vaccination is completely up to me.	Strongly Disagree	833	7.7
	Disagree	613	5.7
	Neutral	2764	25.5
	Agree	2559	23.6
	Strongly Agree	4042	37.3

I have resources, time and opportunities to get a COVID-19 vaccination.	Strongly Disagree	451	4.2
	Disagree	832	7.7
	Neutral	2157	19.9
	Agree	3101	28.6
	Strongly Agree	4265	39.3

I am willing to get a COVID-19 vaccination.	Strongly Disagree	631	5.8
	Disagree	564	5.2
	Neutral	2430	22.4
	Agree	3246	29.9
	Strongly Agree	3932	36.3

I want to get a COVID-19 vaccination.	Strongly Disagree	640	5.9
	Disagree	588	5.4
	Neutral	2624	24.2
	Agree	3198	29.5
	Strongly Agree	3750	34.6

**Table 4:** Distribution of responses on the fear of COVID-19 scale.

Fear of COVID-19 Scale (FCV-19S)		Frequency	Percentages
I am most afraid of Coronavirus-19	Strongly disagree	1181	10.9
	Disagree	1315	12.1
	Neither agree nor disagree	1410	13.0
	Agree	3572	32.9
	Strongly agree	3330	30.7

It makes me uncomfortable to think about Coronavirus-19	Strongly disagree	1020	9.4
	Disagree	1352	12.5
	Neither agree nor disagree	1403	12.9
	Agree	4175	38.5
	Strongly agree	2861	26.4

My hands become clammy when I think about Coronavirus-19	Strongly disagree	3108	28.7
	Disagree	3317	30.6
	Neither agree nor disagree	2221	20.5
	Agree	1340	12.4
	Strongly agree	822	7.6

I am afraid of losing my life because of Coronavirus-19	Strongly disagree	1389	12.8
	Disagree	1330	12.3
	Neither agree nor disagree	1611	14.9
	Agree	3349	30.9
	Strongly agree	3130	28.9

When watching news and stories about Coronavirus-19 on social media, I become nervous or anxious.	Strongly disagree	1509	13.9
	Disagree	1966	18.1
	Neither agree nor disagree	1830	16.9
	Agree	3595	33.2
	Strongly agree	1909	17.6

I cannot sleep because I'm worrying about getting Coronavirus-19	Strongly disagree	3348	30.9
	Disagree	3290	30.3
	Neither agree nor disagree	1812	16.7
	Agree	1507	13.9
	Strongly agree	859	7.9

My heart races or palpitates when I think about getting Coronavirus-19	Strongly disagree	3054	28.2
	Disagree	3357	31.0
	Neither agree nor disagree	1723	15.9
	Agree	1837	16.9
	Strongly agree	836	7.7

**Table 5:** Distribution of responses on the Perceived COVID-19 infectability.

Perceived COVID-19 infectability		Frequency	Percentages
If a COVID-19 patient is “going around”, I will get it	Strongly disagree	2307	21.3
	Disagree	2355	21.7
	Neither agree nor disagree	1913	17.6
	Agree	1997	18.4
	Strongly agree	2234	20.6

My past experiences make me believe I am not likely to get COVID-19 even when my friends are sick	Strongly disagree	1057	9.7
	Disagree	1553	14.3
	Neither agree nor disagree	2448	20.7
	Agree	2827	26.1
	Strongly agree	3120	28.8

In general, I am very susceptible to colds, flu, COVID-19 and other infectious diseases	Strongly disagree	836	7.7
	Disagree	1177	10.9
	Neither agree nor disagree	2320	21.4
	Agree	3191	29.4
	Strongly agree	3282	30.3

I am unlikely to catch a cold, flu, COVID-19 or other illness, even if it is “going around”*	Strongly disagree	2053	18.9
	Disagree	2080	19.2
	Neither agree nor disagree	2691	24.8
	Agree	2452	22.6
	Strongly agree	1527	14.1

My immune system protects me from COVID-19 that other people get*	Strongly disagree	1323	12.2
	Disagree	1455	13.4
	Neither agree nor disagree	1669	15.4
	Agree	2688	24.8
	Strongly agree	3675	33.9

⁎ = Reverse scored.

## 2 Experimental Design, Materials and Methods

The study was carried out using a cross-sectional design with multistage stratified cluster sampling among Qazvin adult residents, which comprised a representative sample of the general population in Qazvin, Iran [12]. Qazvin, a province located in the central part of Iran, is 50 km northwest of Tehran. The 2018 census, which is the latest census, shows that the province has a population of 1,273,761, where 51% were male. The first step of the multistage stratified cluster sampling was to decide six cities as clusters and Qazvin, Takestan, Avaj, Alborz, Buin Zahra, and Abyek were subsequently considered as the present study's clusters. In the second step, each city was stratified according to its urban and rural areas. In the third step, several health centers in each urban and rural areas were randomly selected. In the fourth step, the centers provided a list of families that were covered by their service and the families were randomly selected for participation. In the final step, several interviewers (who received standard training) visited the homes of the selected participants and interviewed the families for this survey. The survey period was between February 19 and April 9, 2021.

The eligibility of the participants depended on fulfilling the following inclusion criteria. First, they had to be an adult resident in Qazvin province who was aged 18 years or above; and second, the participant had to be willing to participate after fully understanding the study's purpose and interview procedure. Moreover, the only exclusion criterion was that participants could not be could either guests or tourists in Iran during the survey period. In order to verify the participants’ willingness to participate, each participant provided a written Informed consent. All the data were analyzed using descriptive statics (i.e., mean with SD; frequency with percentage) and internal consistency (i.e., Cronbach's α) carried out by the IBM SPSS 17.0.

Fear of COVID-19 was assessed using the 7-item Fear of COVID-19 Scale (FCV-19S), where all the items were rated on a 5-point Likert scale [13]; the FCV-19S had satisfactory psychometric properties in the present dataset (internal consistency *α* = 0.88). Perceived COVID-19 infectability was assessed using 5 items rated on a 5-point Likert scale; the 5 items had satisfactory psychometric properties in the present dataset (internal consistency *α* = 0.70). Perceived behavioral control over COVID-19 vaccination was assessed using 2 items rated on a 5-point Likert scale; the 2 items had satisfactory psychometric properties in the present dataset (internal consistency *α* = 0.75). Subjective norm of COVID-19 vaccination was assessed using 2 items rated on a 5-point Likert scale; the 2 items had satisfactory psychometric properties in the present dataset (internal consistency *α =* 0.89). Attitude towards COVID-19 vaccination was assessed using 6 items rated on a 5-point Likert scale; the 6 items had satisfactory psychometric properties in the present dataset (internal consistency *α =* 0.94). Intention to get COVID-19 vaccinated was assessed using 2 items rated on a 5-point Likert scale; the 2 items had satisfactory psychometric properties in the present dataset (internal consistency *α =* 0.92).

## Ethics Statement

In collecting the data, the 1975 Helsinki declaration and ethical permission to collect the data was granted from the Ethics Committee of Qazvin University of Medical Sciences (protocol code: IR.QUMS.REC.1399.418; date of approval: 20 January 2021). Additionally, written informed consent was provided by all participants prior to starting the survey. They were informed about the purpose and nature of the data and they had the right to withdraw their data if they wanted to.

## CRediT authorship contribution statement

**Amir H. Pakpour:** Conceptualization, Investigation, Writing – original draft, Funding acquisition, Formal analysis, Supervision. **Rafat Yahaghi:** Data curation. **Safie Ahmadizade:** Data curation. **Razie Fotuhi:** Data curation. **Elham Taherkhani:** Data curation. **Mehdi Ranjbaran:** Data curation. **Zeinab Buchali:** Data curation. **Chung-Ying Lin:** Investigation, Writing – original draft, Formal analysis. **Mark D. Griffiths:** Writing – original draft. **Anders Broström:** Data curation, Investigation.

## Declaration of Competing Interest

The authors declare that they have no known competing financial interests or personal relationships that could have appeared to influence the work reported in this paper.

## Appendix Supplementary materials

Image, application 1
