# Supplementary material for: A population-based dataset concerning predictors of willingness to get a COVID-19 vaccine in Iran
Source: Data Brief. 2021 Oct 8;39:107459. doi: 10.1016/j.dib.2021.107459 (PMC8500683; doi:10.1016/j.dib.2021.107459)
Supplement: Supplementary file 1 [file mmc1.docx]

***Predictors of willingness to get a COVID-19 vaccine in Iran***

***Consent***

**Do you want to participate in this survey?**

1. Yes
2. No

***Socio-demographics (SD)***

**SD_1: Your age in years (write in number, e.g., 20)**

…………………………..

**SD_2: Gender**

1. Male
2. Female

**SD_3: Educational qualification**

1. No formal education
2. Primary school
3. Secondary school
4. High school
5. Diploma
6. University

**SD_5: If you currently a *student*, what is your discipline?**

1. Pure science
2. Medical or allied health science
3. Arts or social science
4. Engineering
5. Business studies
6. Others

**SD_6: Current place of residence (e.g., Dhaka district)**

…………………………..

**SD_7: Which type of administrative region are you living in?**

1. City
2. Rural

**SD_8: Marital status**

1. Single
2. Married

**SD_12: do you have any child (under 8 years)**

1. Yes
2. No

***Attitude toward COVID-19 vaccination***

**For me, getting the COVID-19 vaccination would be …**

1. extremely bad (1) (2) (3) (4) extremely good (5)
2. extremely undesirable (1) (2) (3) (4) extremely desirable (5)
3. extremely unimportant (1) (2) (3) (4) extremely important (5)
4. extremely useless (1) (2) (3) (4) extremely useful (5)
5. extremely unfavorable (1) (2) (3) (4) extremely favorable (5)
6. extremely harmful (1) (2) (3) (4) extremely beneficial (5)

***Subjective norms***

**Answers included: 1. Strongly disagree 2. Disagree 3. Neither agree nor disagree 4. Agree 5. Strongly agree**

**Most people who are important to me would want me to get COVID-19 vaccination**

**Most people who are important to me would think I should get COVID-19 vaccination**

**Perceived Behavioral Control**

**Answers included: 1. Strongly disagree 2. Disagree 3. Neither agree nor disagree 4. Agree 5. Strongly agree**

**Whether or not I get COVID-19 vaccination is completely up to me.**

**I have resources, time and opportunities to get COVID-19 vaccination.**

**Intention**

**Answers included: 1. Strongly disagree 2. Disagree 3. Neither agree nor disagree 4. Agree 5. Strongly agree**

**I am willing to get COVID-19 vaccination.**

**I want to get COVID-19 vaccination.**

**Fear of COVID-19 Scale (FCV-19S)**

**Answers included: 1. Strongly disagree 2. Disagree 3. Neither agree nor disagree 4. Agree 5. Strongly agree**

**FCV-19S_1:** I am most afraid of Coronavirus-19

**FCV-19S_2:** It makes me uncomfortable to think about Coronavirus-19

**FCV-19S_3:** My hands become clammy when I think about Coronavirus-19

**FCV-19S_4:** I am afraid of losing my life because of Coronavirus-19

**FCV-19S_5:** When watching news and stories about Coronavirus-19 on social media, I become nervous or anxious.

**FCV-19S_6:** I cannot sleep because I’m worrying about getting Coronavirus-19

**FCV-19S_7:** My heart races or palpitates when I think about getting Coronavirus-19

**Perceived COVID-19 infectability**

**Answers included: 1. Strongly disagree 2. Disagree 3. Neither agree nor disagree 4. Agree 5. Strongly agree**

If a COVID-19 patient is “going around”, I will get it

My past experiences make me believe I am not likely to get COVID-19 even when my friends are sick

In general, I am very susceptible to colds, flu, COVID-19 and other infectious diseases

I am unlikely to catch a cold, flu, COVID-19 or other illness, even if it is “going around”

My immune system protects me from COVID-19 that other people get
